# Inflammatory profile of keratoconic corneal epithelium

**DOI:** 10.1186/s12886-023-03013-0

**Published:** 2023-07-17

**Authors:** Junia Cabral Marques, Karina Inácio Ladislau de Carvalho, Rafaela Xavier, Walton Nosé, Luiz Vicente Rizzo

**Affiliations:** 1 Hospital Israelita Albert Einstein, Instituto Israelita de Ensino e Pesquisa, São Paulo, Brazil; 2 Federal University of São Paulo, São Paulo, Brazil

**Keywords:** Keratoconus, Neuropeptides, Chemokines, Inflammation, Cornea

## Abstract

**Background:**

Recent studies have presented inflammatory features on keratoconus (KC) and many inflammatory markers are described in the tears of patients with this disease. The KC pathogenesis is still unknown just like the correlation with inflammatory patterns. However, environmental and genetic issues may be part of the progress of KC. In addition, some systemic features, such as allergy and obesity, seem to be related to the progression of KC. Our purpose was to evaluate the neuropeptides vasoactive intestinal peptide (VIP), neuropeptide Y (NPY), chemokines ligand 2 (CCL-2) and 5 (CCL-5), and interleukins 6 (IL-6) and 8 (IL-8) on corneal epithelial cells and blood of patients with KC and in healthy controls. In addition, the neutrophil-to-lymphocyte ratio (NLR) was evaluated to predict inflammation.

**Methods:**

This including prospective observational study included 32 KC patients who underwent corneal crosslinking (CXL) and 32 control patients who underwent photorefractive keratectomy (PRK). Patients’ corneal epithelial cells were removed surgically, and blood (buffy coat) was analyzed. Samples in triplicate were evaluated on rt-PCR for neuropeptides (VIP e NPY), interleukins (IL-6 e IL-8), and chemokines (CCL-2 and CCL-5).

**Results:**

Our study showed statistically higher CCL-5 and IL-8 on corneal epithelial cells in patients with KC. Blood cells were statistically higher in VIP and NPY in the KC group. Interleukin-8 on blood cells was statistically significant in KC’S group; for CCL-2 and CCL-5 they were statistically lower in patients with KC compared with controls. NLR showed no difference between the groups.

**Conclusions:**

Our data support the findings of other studies that suggested altering KC status, such as inflammatory corneal disease. The presence of IL-8 in the cornea and blood samples of KC’s group suggested systemic disease with a possible local or repercussion action. Further studies are warranted to elucidate KC pathogenesis and its correlation to systemic disease.

## Introduction

Keratoconus (KC) is an ocular disease characterized by the steepening and thinning of the cornea that may lead to visual impairment [1]. The pathogenesis of this disease is not well established, and many studies have highlighted the presence of inflammatory status of the cornea [2–7]. Proteases, cytokines, tumor necrosis factor-alpha and beta, and proteolytic activity are described in tear film of patients with KC [8–12].

Peripheral blood samples have been studied to correlate KC with other systemic changes linked to inflammation [2]. Neutrophil-to-lymphocyte ratio has been used as a marker of inflammation, and it has been associated with the severity and prognosis of some cardiologic and oncologic diseases. In addition, this ratio has been described in ophthalmological diseases, and this incidence has been found to be raised in patients with KC [13].

Neuropeptides are molecules produced and released by neurons. They have a trophic effect communicating neurons and effector cells like glands, muscles, and immune cells [14]. Some authors defend that corneal innervation releases neuropeptides on the ocular surface and then they active immune system leading to neurogenic inflammation [15–17]. Mostly studied neuropeptides in ocular diseases are CGRP, SP, VIP, and NPY.

Interleukins (IL) and chemokines are small molecules involved in the cells immune response mediating some physiological signaling like allergy and inflammation. A disbalance between proinflammatory and anti-inflammatory molecules may alter local homeostasis and elevate proteolytic activity, associated with KC progression [18].

This study’s aim was to evaluate neuropeptides VIP and NPY, chemokines CCL-2 and CCL-5, and interleukins IL-6 and IL-8 on corneal epithelial cells and blood, in patients with KC and health controls. In addition, we also studied neutrophil-to-lymphocyte ratio (NLR) to predict inflammation.

## Materials and methods

Thirty-two eyes previously diagnosed with KC that would be submitted to corneal crosslinking (CXL) and thirty-two normal eyes that underwent photorefractive keratectomy (PRK) were enrolled in the study. All patients accepted the consent form that was previously approved by the ethics committee of the Hospital Israelita Albert Einstein (São Paulo, SP, Brazil) in accordance with the Declaration of Helsinki.

Both groups were asked about their height and weight and also about the continuous use of medication or systemic diseases. Patients using topical or systemic corticosteroids or non-steroid medication, and antiallergic or immunosuppressive drugs were not eligible. All participants were not previously submitted ocular surgeries.

Blood samples were collected before corneal samples for all subjects. And then, they were immediately processed. Buffy coat was separated and placed in the freezer at -70 °C. Corneal epithelium samples were collected with a crescent blade and stored on RNA later in the freezer at -70 °C. We extracted cDNA from all samples using PureLink ™ DNAse (ThermoFisher Scientific) and then thermal cycler according to manufacturer’s instructions.

Real-time PCR QuantStudio 6 Flex Real-Time PCR System® (Applied BioSystems, ThermoFisher Sc.) was then performed with Power SYBR Green PCR Master Mix (Applied Biosystems, Foster City, CA, EUA). The primers (Invitrogen®, ThermoFisher Sc.) in triplicate we used were: *Glyceraldehyde 3-phosphate dehydrogenase* (GAPDH) as a control; *Vasoactive Intestinal Neuropeptide* – VIP; *Neuropeptide Y* – NPY; *Interleukin-6* - IL-6; *Interleukin − 8* - IL-8; *Chemokine ligand 2* - CCL2; *Chemokine ligand 5* - CCL5. The system ran hold stage 95℃ for 10 min; PCR stage (40 cycles) -95℃ for 15 s, followed by 60℃ for 1 min; *Melt curve stage −* 95℃ for 15 s, and then 60℃ for 1 min and 95℃ for 15 s.

### Statistics

Fisher’s exact or chi-square tests were adopted for qualitative data analysis. Quantitative data were performed with histograms and quartiles, and Shapiro-Wilk test was also performed to test normality. Samples were described using mean and standard deviation. Quantitative data comparison between groups was performed using the Student’s t-test and by adopting normality and Mann-Whitney test for other data. Spearman correlation was performed to evaluate correlation between quantitative data. All the analyses were performed by SPSS software (IBM Corp. IBM SPSS Statistics for Windows, Version 26.0. 2019) adopting statistics significance of 5%.

## Results

We had 64 eyes divided into two groups: 32 eyes with KC and 32 normal controls. Men were 62.5% in the KC group and 53.1% in the control group (p = 0.448). The mean age was 26.3 in the KC group and 27.5 years (p = 0.309) in the control group. In terms of height no difference was noticed between groups (p = 0,192); however, there was a statistical difference in weight and body mass index (BMI) in KC group (p = 0.007 and p = 0.015, respectively). Considering overweight, we found 71.9% in KC group and 37.5% in the control group (p = 0.006). This is the first description of the association between KC and obesity in Brazil.

The right eye was found more frequent in the control group (53.1%) than in the KC group (43.8%) with p-value 0,453. The presence of other diseases was more frequent in the KC group (37.5%) and the most commonly cited disease was respiratory allergy (p = 0.604) followed by thyroids disease (p = 0.450) and anxiety or depression (p > 0.99). The continuous use of medication was cited by 43.8% of KC group and 21.9% of controls (p = 0.062). Table 1 describes the sociodemographic characteristics of both groups.

**Table 1:** Sociodemographic and clinical features of groups

	**Group**		p-Value
	Control (n = 32)	**KC (n = 32)**	
Genre n (%)			
Female	15 (46.9)	12 (37.5)	
Male	17 (53.1)	20 (62.5)	
Age (at date of data collection)			0.309 tc
Mean (standard deviation)	27.5 (2.6)	26.3 (5.7)	
Weight (kg)			0.007 m
Mean (standard deviation)	72.5 (17.9)	84.2 (17.7)	
Height (m)			0.192 t
Mean (standard deviation)	169.4 (10.3)	173.1 (12.0)	
BMI			0.15 m
Mean (standard deviation)	25.0 (4.5)	28.1 95.6)	
Laterality n (%)			0.453 q
Right eye	20 (62.5)		0.006 q
Left eye	12 (37.5)	14 (43.8)	
Overweight or obesity n (%)		18 (56.3)	
No (BMI < 25)			
Yes (BMI≥25)		9 (28.1)	
		23 (71.9)	
Presence of systemic disease n (%)	28 (87.5)	20 (62.5)	0.021 q
No	4 (12.5)	12 (37.5)	
Yes			
Thyroid disease n (%)	3 (75)	11 (91.7)	0.450 f
No	1 (25)	1 (8.3)	
Yes			
Respiratory allergy n(%)	2 (50)	4 (33.3)	0.604 f
No	2 (50)	8 (66.7)	
Yes			
Maximum keratometry	44.0 (1.6)	55.2 (5.8)	<0.001 µ
Mean (standard deviation)			
Minimum pachymetry (µm)	531.2 (34.6)	487.9 (40.1)	<0.001 τ
Mean (standard deviation)			

KC: keratoconus; BMI: body mass index; f: Fisher test; q: chi-square test; tc: t test for unequal variable; t: *t test of* Student; m: Mann-Whitney test

The lymphocytes, neutrophils, and NLR index are described in Table 2. There was no statistical difference between groups on NLR blood samples demonstrated as follows in Table 2.

**Table 2:** Blood samples results

Blood samples	Group		p-Value
	Control	KC	
Lymphocytes (%)			0,727 m
Mean (standard deviation)	39.7 (35.4)	32.7 (7.7)	
Lymphocytes counting			0.304 m
Mean (standard deviation)	4.2 (8.9)	2.4 (0.7)	
Neutrophils (%)			0.638 m
Mean (standard deviation)	55.5 (10.8)	56.0 (8.5)	
Neutrophils counting			0.456 m
Mean (standard deviation)	4.4 (1.5)	4.2 (1.3)	
NLR			0.995 m
Mean (standard deviation)	1.7 (0.6)	1.9 (1.1)	

KC: keratoconus; NLR: neutrophil/lymphocyte ratio; t: *t test of* Student; m: Mann-Whitney test

Comparison between corneal samples and buffy coat on different genes of interest.

All samples were analyzed in fold induction for cornea (C) and blood samples (buffy coat – BC), as shown in Table 3.

On corneal samples, neuropeptides VIP and NPY did not show the statistical difference (respectively p = 0.413 and p = 0,638). Chemokine CCL-2 was p = 0.052, and CCL-5 was p < 0.001. Interleukin IL-8 was p = 0.002 while IL-6 presented no statistical significance (p = 0.376).

For BC results both VIP and NPY were statistically different (p < 0.001). Chemokines CCL-2 and CCL-5 showed statistical difference results in the KC group (respectively p = 0.006 and p = 0.005). As seen on corneal results, BC has a statistical difference on IL-8 (p < 0.001) but not on IL-6 (p = 0.063).

The IL-8 is augmented on the KC group on corneal epithelia and BC.

**Table 3:** Results on fold induction

	Group		p-Value
	Control	KC	
VIP corneal epithelium	1.0 (0.0; 8.2)	0.3 (0.0; 9.4)	
Median (1 º, 3 º quartile)			0.413 m
NPY corneal epithelium			0.638 m
Median (1º; 3º quartile)	1.0 (0.4; 8.0)	1.1 (0.5; 13.6)	
CCL-2 corneal epithelium			0.052 m
Median (1º; 3º quartile)	1.0 (0.4; 2.2)	0.2 (0.1; 1.8)	
CCL-5 corneal epithelium			< 0.001 m
Median (1º; 3º quartile)	1.0 (0.3; 3.5)	20161.8 (9983.5; 55364.2)	
IL-8 corneal epithelium			0.002 m
Median (1º; 3º quartile)	1.0 (0.5; 17.6)	89.0 (3.5; 423.0)	
IL-6 corneal epithelium			0.376 m
Median (1º; 3º quartile)	1.0 (0.4; 2.2)	0.7 (0.3; 1.5)	
VIP BC			< 0.001 m
Median (1º; 3º quartile)	1.0 (0.1; 8.6)	64.2 (5.1; 440.4)	
NPY BC			< 0.001 m
Median (1º; 3º quartile)	1.0 (0.8; 1.5)	24.6 (16.6; 33.3)	
CCL-2 BC			0.006 m
Median (1º; 3º quartile)	1.0 (0.3; 2.0)	0.2 (0.0; 0.9)	
CCL-5 BC			0.005 m
Median (1º; 3º quartile)	1.0 (0.4; 7.4)	0.3 (0.2; 2.3)	
IL-8 BC			< 0.001 m
Median (1º; 3º quartile)	1.0 (0.4; 4.0)	29.4 (18.8; 166.4)	
IL-6 BC			0.063 m
Median (1º; 3º quartile)	1.0 (0.3; 10.2)	0.5 (0.2; 1.3)	

BC: *buffy coat*.; CCL: chemokine ligand CC; IL: interleukin; NPY: neuropeptide Y; VIP: intestinal vasoactive peptide; m: Mann-Whitney test

## Discussion

Keratoconus has still unknown etiology, which is possibly associated with environmental, biomechanics, biochemical, and genetic factors, or maybe the association between them. Studies show no genre predilection, since they have methodological differences [1, 18–20]. Our study showed more male individuals in both groups with no statistical significance as reported by other studies in the published literature.

Our results demonstrated that in our sample patients with KC are associated with higher overweight and obesity than those in the control group. We believe it is the first description in Brazilian literature correlates obesity and KC. This correlation is described in the literature, although it’s not well established [21]. Obese individuals have higher incidences of inflammation, a chance of infection, and other comorbidities [22]. Recently an Israeli study with adolescents showed higher chances of developing kc among those who are overweight or obese [23]. However, a Turkish study demonstrated no statistical difference in body mass index (BMI) between KC and the control healthy group [24]. It is believed that obesity could be a risk factor for development of KC in predisposed people and this might occur due to decreased tarsal elastin, which leads to laxity of palpebral skin, changes palpebral function, and provides corneal irritation [21]. Also, a pro-inflammatory environment with decreased immune response is associated with adipose tissue accumulation [22] and could be correlated with KC pathogenesis.

Our study described the presence of other diseases in the KC group compared with controls. Claessens et al. [25] discuss the positive association of KC and immune-mediated diseases. Among them are respiratory allergies and psychiatric diseases (depression and anxiety). Allergy was not statically significant in our study as shown by Woodward et al., 2016 [26]. However, it is often described among KC patients and might be poorly diagnosed because of a rare clinical impact that does not require continuous treatment. This may justify the fact that KC patient’s rubber eyes is associated with disease progression [1] and allergy [26, 27]. Lema et al. described that 64.3% of KC patients reported that they rub their eyes frequently and vigorously [10]. Sharma et al. showed a strong correlation between ocular allergy and KC progression [4]. It is important to remember that inflammatory events lead to biochemical events and accumulation of immune cells that might cause local discomfort and consequentially itching.

Tears and corneal samples are wildly used in KC research, and since systemic repercussion is not well established in KC, some studies described blood investigation.

The neutrophil-to-lymphocyte ratio (NLR) shows a number that might be used as an inflammation predictor [13]. This author described statistical results in the KC patients, the same showed by Oltulu et al. [28]. Otherwise, our results agree with Bozkurt [29], that did not show a difference between groups on NLR.

We also investigated neuropeptides which participate in the wound healing process and homeostasis [14, 30]. These molecules are liberated by neurons that innervate the cornea. We studied VIP and NPY peptides in cornea and blood samples. In corneal epithelium, we did not find statistical significance for both neuropeptides, unlike Sachetti et al. [31], which found VIP higher in corneal tissue from keratoplasty of KC patients. Our results showed that VIP and NPY were higher in KC patients’ blood samples. We did not find any research correlating blood samples, neuropeptides and KC. However, studies in mice with obesity showed NPY concentration on blood was found to be higher. The NPY can be produced by adipocyte cells and on fatty tissue might participate as an autocrine, paracrine, or endocrine mediator. This peptide also promotes adipocyte differentiation and fat accumulation in white tissue metabolism [32].

In our corneal epithelium samples, no statistical difference was found on neuropeptides. However, neuropeptide VIP was described by Sacchetti el al. but in corneal tissue from keratoplasty [31]. The VIP is known to have anti-inflammatory action inhibiting pro-inflammatory chemokines like CCL-2 and 5 [17]. Mantelli et al. described that neuropeptides could be enrolled in local inflammation by neurogenic inflammation, in which a stimulus liberates local neuromodulators and produce and promotes local liberation of cytokines and chemokines [16].

The chemokines regulate inflammatory response and homeostasis [33]. We demonstrate that CCL-2 was higher expressed in the control group for both corneal and blood samples. Ebihara et al. described a possible association of ocular inflammation conditions with the expression of CCL-2 on normal corneal epithelium cells and in a culture of keratocytes cells and their receptor interaction on corneal layers [34]. Recently, Villarreal-Ponce et al. described the influence of CCL-2 in corneal healing that suggested that its absence delays wound healing [35]. We then infer that lower levels of CCL-2 could affect the wound-healing process in corneal KC patients.

The CCL-5 augmented on KC patients may suggest local inflammation once this chemokine is related to many other inflammatory situations like angiogenesis, cancer, upper airway, and rheumatoid arthritis [36]. Previous studies have described CCL-5 in tears [6], but not on corneal tissue. To the best of our knowledge, this is the first study to describe it.

Chemokines and interleukins are described in KC tears samples [9, 10, 28]. Interleukin 6 is one of the studied molecules in KC tears. It regulates MMP-9 that acts in the corneal collagen structure matrix that might play a role in KC pathogenesis [18]. Taurone et al. [37] found statistically higher IL-6 on immunohistochemical essay of keratoconic corneal tissue. However, in our study, no statistical difference was found in corneal or blood samples. The IL-6 acts on the inflammation cascade and induces CCL-2 and IL-8 expression [38].

In comparison with Spandau et al. [39] our study showed IL-8 on corneal cells in both groups; however, which was statistically augmented in KC group. Interleukin-8 is synthesized by corneal cells as a local inflammation response [12] and it was also described as a component of allergic response [40]. Higher concentrations of IL-8 in the cornea and blood of KC patients allow us to infer a correlation with a systemic disease with influence on the eyes. Obesity could be, among others, a stimulus for inflammation and/or reason to perpetuate (or be redundant) IL-8 action [41, 42].

In conclusion, our data support findings of previous studies that suggested the alteration of KC status such as inflammatory corneal disease. The presence of IL-8 in the cornea and blood samples from KC’s group suggested a correlation with a systemic disease with a possible local or repercussion action. Further studies must elucidate KC pathogenesis and its correlation with systemic disease.

## Data Availability

The datasets used and analyzed during the current study are available from corresponding author upon reasonable request.

## Abbreviations

BC: Buffy coat
CCL: Chemokine ligand
CGRP: Calcitonin-gene-related peptide
CXL: Corneal colagen crosslinking
DEPC: Diethyl pyrocarbonate
GADPH: Glyceraldehyde 3-phosphate dehydrogenase
IL: Interleukin
KC: Keratoconus
MMP: Metilmetaloproteinase
NLR: Neutrophil-to-lymphocyte ratio
NPY: Neuropeptide Y
PCR: Polimerase chain reaction
PRK: Photorefractive keratectomy
SP: Substance P
TGF: Transforming growth factor
TNF: Tumoral necrosis factor
VIP: Vasoative intestinal peptide
